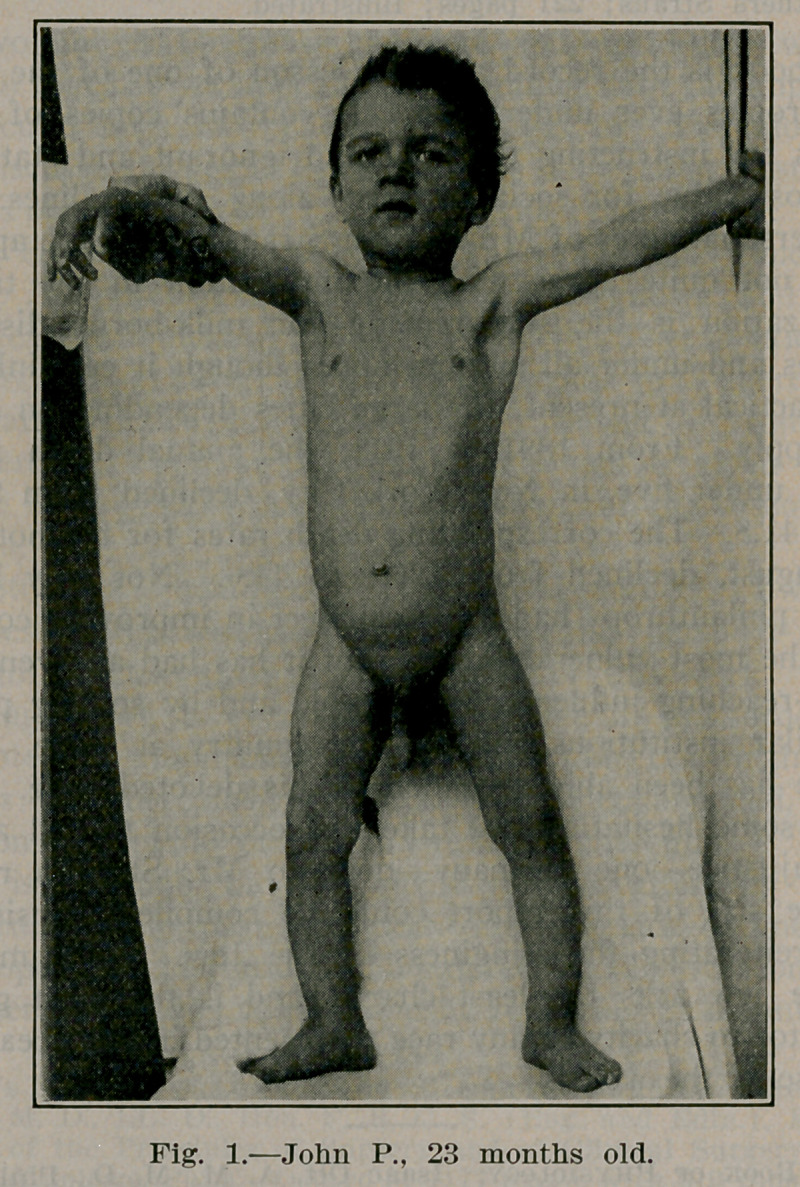# Abnormal Development of Two-Year-Old Child

**Published:** 1913-05

**Authors:** 


### ABSTRACTS.

Abnormal Development of Two-Year-Old Child. John Lovett Morse, Boston, *Arch. of Paediatrics,* March, 1913. (Cut kindly loaned by journal.) There was no excess of hair on the face. The penis was very large, measuring 7½ cm. in length when flaccid and 10cm. when erect. The testicles were the size of robin's eggs. He showed no evidence of sexual feel-

Fig. 1.—John P., 23 months old.

ing and repeated examinations of the centrifugalized urinary sediment failed to show spermatozoa.

| Measurements. | John P. | Normal at 2 years. | Normal at 4 years. |
|---|---|---|---|
| Body length, crown to sole...... | 98 cm. | 82.8 cm. | 96.7 cm. |
| Circumference of head......... | 52 cm. | 48.2 cm. | 50.3 cm. |
| Circumference of chest......... | 54 cm. | 48.4 cm. | 52.8 cm. |
| Weight ...................... | 35 lbs. | 26.5 lbs. | 35 lbs. |

Five other male cases are collected from the literature, in one of which there was pressure atrophy of the pituitary. The adrenals were normal in two, contained nodular tumors and showed superactive cortex and medulla in one. All showed pineal gland tumor, whose characteristic symptoms are stated by Glynn to be those of brain tumor in general plus precocious sexual development.

## Figures and Tables

**Fig. 1. f1:**